# METAMORPHOSES: THE CONTINUING EVOLUTION AND ADAPTATION OF THE SAVVY CAREGIVER PROGRAM

**DOI:** 10.1093/geroni/igac059.1699

**Published:** 2022-12-20

**Authors:** Kenneth Hepburn, Joseph Gaugler

## Abstract

This symposium traces the nearly 30 years of development projects, trials, and transformations that continue to occur in efforts to make an evidence-based psychoeducation program (Savvy Caregiver) available to family members and friends who provide care to community-dwelling persons living with dementia illnesses. Savvy seeks to enhance caregivers’ knowledge and skills for providing care and to enhance their competence and confidence (sense of mastery) in being able to guide their persons through days that are as safe, calm, and pleasant as possible. Over this period of time, Savvy has clarified its target participant group (focusing on primary caregivers rather than larger family groups), moved from having concurrent sessions for caregivers and persons living with dementia, continued to develop strategies that enable broad implementation while maintaining program fidelity, demonstrated the effectiveness of a synchronous/asynchronous online version that extends program reach (Tele-Savvy), and is currently exploring in an ongoing pilot how best to deliver a fully asynchronous version of the program that employs continuing education principles (Tele-Savvy@Home). The individual presentations will describe the main developmental milestones (investigator led to protocol-trained to online trained interventionist; in-person to synchronous/asynchronous to fully asynchronous) and the structural adaptations that enabled them. Particular emphasis will be placed on design decisions linked to maintaining a psychoeducational orientation and to program emphasis on developing or strengthening caregivers’ self-efficacy for providing effective day-to-day care. Savvy’s experience may help investigators in designing new interventions, particularly online interventions or in considering augmentation or delivery platform change of established interventions.

